# P04.07. Patients’ reasons for use of traditional East Asian medicine as an alternative treatment

**DOI:** 10.1186/1472-6882-12-S1-P277

**Published:** 2012-06-12

**Authors:** A Burke, E Herlambang

**Affiliations:** 1 San Francisco State University, San Francisco, USA

## Purpose

In an effort to better understand why individuals use complementary and alternative medicine a single whole systems provider-based modality was selected, one with the potential for use as an alternative to allopathic care for a broad array of conditions -- Traditional East Asian Medicine (TEAM). A survey was conducted with a sample of individuals who had used TEAM examining their motives for use and perceived outcomes in relation to allopathic medical care options.

## Methods

Data was collected from a convenience sample of 222 participants using an 88-item survey. Variables included frequency of use, health status, personal experience with acupuncture and allopathic care, satisfaction and perceived efficacy.

## Results

The most commonly cited reasons for use were musculoskeletal conditions, followed by gynecological and gastrointestinal. For the majority (62%) this presenting complaint was a chronic condition. When asked how their decision to use acupuncture related to the use of allopathic medical care the most common response (37.5%) was, “I felt that allopathic medicine was not helping, or could not help me.” The majority (61.5%) reported having seen an allopathic provider for the presenting complaint, and nearly half of them were still seeing an allopathic provider for the problem (43.3%). Of those who were relying on TEAM exclusively the most common reason given (28.3%) was, “The treatments (allopathic) were not working for me.” When asked about the perceived efficacy of the two treatments participants were significantly more likely to report that TEAM had helped them manage their health concern. Multiple regression analyses on key outcome variables found perceived efficacy of acupuncture to be one of the strongest predictors of current and intended future use.

## Conclusion

A primary motive for use appears to be the need for alternatives to allopathic care for unremitting health issues. This suggests a practical problem solving orientation by consumers to healthcare decision making.

